# First application of the varipulse™ catheter for linear ablation in atrial fibrillation: a case report

**DOI:** 10.3389/fcvm.2025.1699236

**Published:** 2025-12-15

**Authors:** Cheng Li, Xia Yu, Hu Zhicheng, Han Lei, Zhang Tao, Xiong Yulong, Yao Yan, Ding Ligang

**Affiliations:** 1 Department of Cardiology, Peoples’ Hospital of Chongqing Liang Jiang New Area, Chongqing, China; 2 Cardiac Arrhythmia Center, National Center for Cardiovascular Diseases, National Key Laboratory, Chinese Academy of Medical Sciences and Peking Union Medical College, Fuwai Hospital, Beijing, China

**Keywords:** atrial fibrillation, linear ablation, pulmonary vein isolation, pulsed field ablation, the varipulse™ catheter

## Abstract

**Background:**

This case report documents the application of the Varipulse™ catheter in linear ablation, offering a successful exploratory experience for the linear ablation of atrial fibrillation.

**Case presentation:**

The patient exhibited signs of AF. After successful completion of pulmonary vein potential isolation and mitral isthmus line ablation, atrial fibrillation was converted into atrial flutter. Integrating the atrial flutter's activation sequence with coronary sinus electrode mapping, we localized the reentrant circuit to the tricuspid isthmus and the surgical incision. Targeted ablation was sequentially performed at these identified sites, and subsequently, the patient's rhythm converted to sinus rhythm.

**Discussion:**

Linear ablation for AF faces substantial technical challenges in regions with complex anatomy, the Varipulse™ catheter is applicable with proven favorable reliability and safety profiles.

## Graphical Abstract

**Figure:**
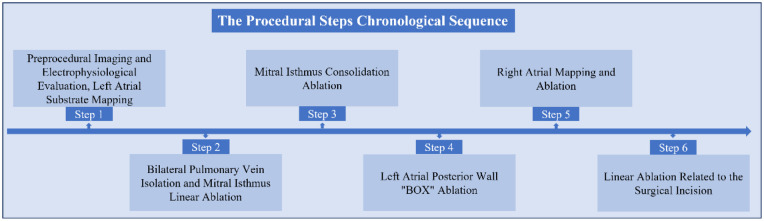
The Procedural Steps in Chronological Sequence.

## Introduction

Pulsed field ablation (PFA) is a recently developed technology that induces irreversible electroporation-mediated tissue necrosis and has been employed and validated for pulmonary vein isolation (PVI) in atrial fibrillation (AF) ablation (1, 2). To date, limited PFA devices have obtained CE Mark certification for this use and the Varipulse™ catheter (Biosense Webster Inc., Irvine, CA, USA), a variable loop PFA catheter, has been recently approved (3). Despite the growing experience with PVI, evidence is lacking about the use of PFA catheters and linear ablation lesions, which could potentially be performed with PFA catheters, reducing procedure related costs and time (4–7). This case report documents the first application of the Varipulse™ catheter in linear ablation, offering a successful exploratory experience for the linear ablation of atrial fibrillation, and demonstrating the feasibility and acute procedural safety of linear ablation. The technique of the Varipulse™ catheter adhesion at the tricuspid isthmus was presented for the first time.

## Case presentation

A 36-year-old female patient was diagnosed with an atrial septal defect (ASD) 10 years ago and underwent patch closure of the ASD via a right atriotomy. Recently, she suffered an acute cerebral infarction due to paroxysmal atrial fibrillation and received thrombolysis at a local hospital, fortunately with no residual symptoms. In pre-hospital examination, the ECG indicated sinus bradycardia, complete right bundle branch block, and ST-T changes, and the pulmonary vein CTA showed the morphology of pulmonary veins. The risk of thromboembolism and bleeding was stratified using the CHA₂DS₂-VASc and HAS-BLED scores, respectively. The CHA₂DS₂-VASc score was 3 (assigning 1 point for female sex and 2 points for a history of stroke), indicating a moderate risk. The HAS-BLED score was 1 (assigning 1 point for a history of stroke), suggesting a low bleeding risk. The patient's baseline echocardiographic findings, a left atrial diameter of 38 mm, a left atrial volume of 48 mL, and a preserved left ventricular ejection fraction (LVEF) of 65%. Furthermore, no residual shunt was observed after the prior atrial septal defect repair. Prior to the stroke onset, the patient was not on any anticoagulant therapy. Following the event, the regimen was initiated with dabigatran etexilate 110 mg twice daily, without any concomitant antiarrhythmic drugs.

**Figure 1:**
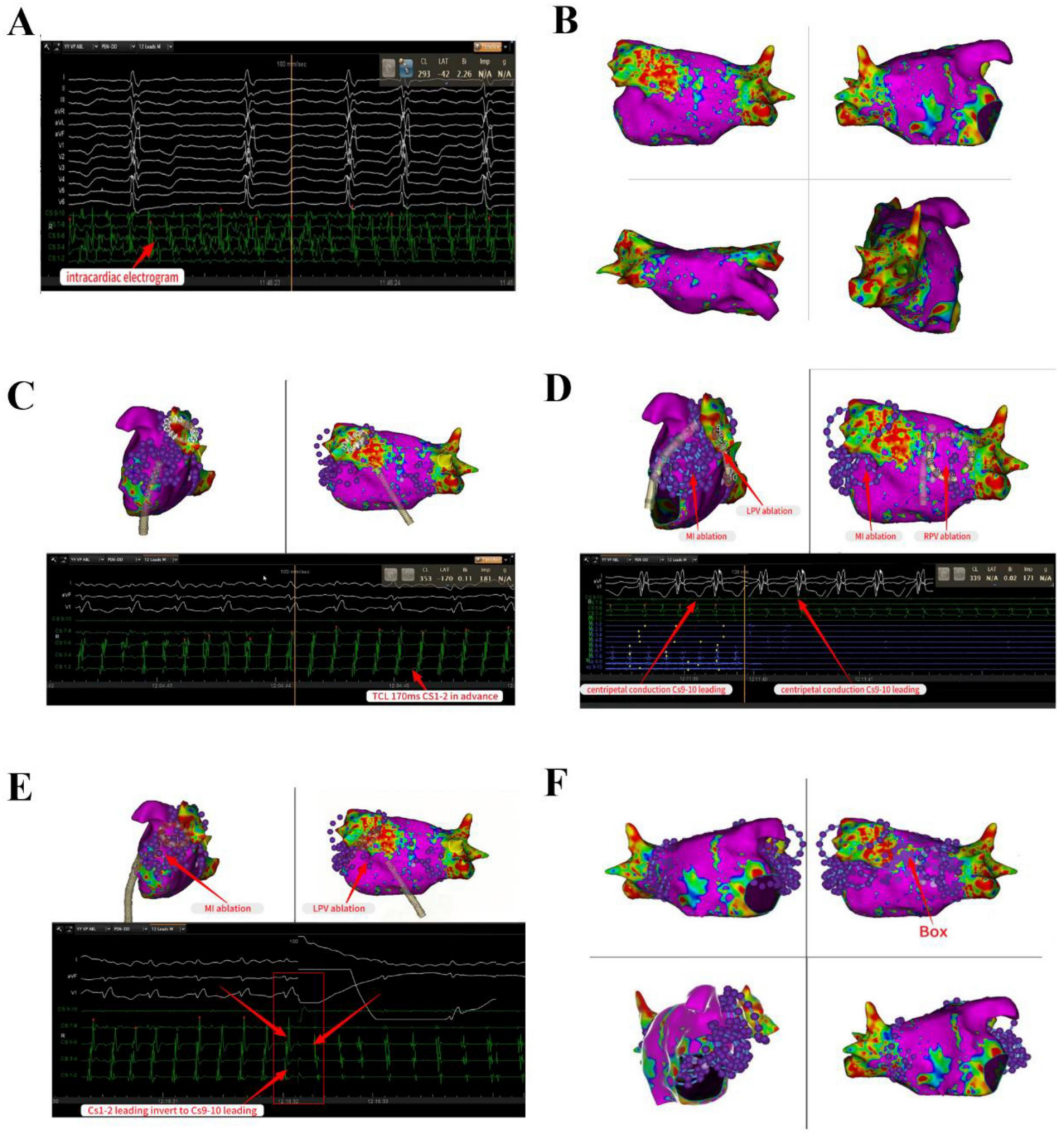
PFA for pulmonary vein isolation and linear ablation in the left atrial intracardiac electrocardiogram shows AF. Left atrial substrate mapping while the AF remained ongoing **(A,B)**. Successful completion of PV potential isolation and mitral isthmus line ablation, AF was converted into atrial flutter with a cycle length of 170 ms and demonstrated eccentric conduction with early CS1-2 polar atrial waves **(C)**. Reinforcing linear ablation of the mitral isthmus, the atrial flutter presented a cycle length of 210 ms and exhibited eccentric conduction marked by early CS9-10 polar atrial waves **(D,E)**. Perform BOX ablation on the posterior wall of the left atrial **(F)**. AF, atrial fibrillation; PV, pulmonary vein.

**Figure 2:**
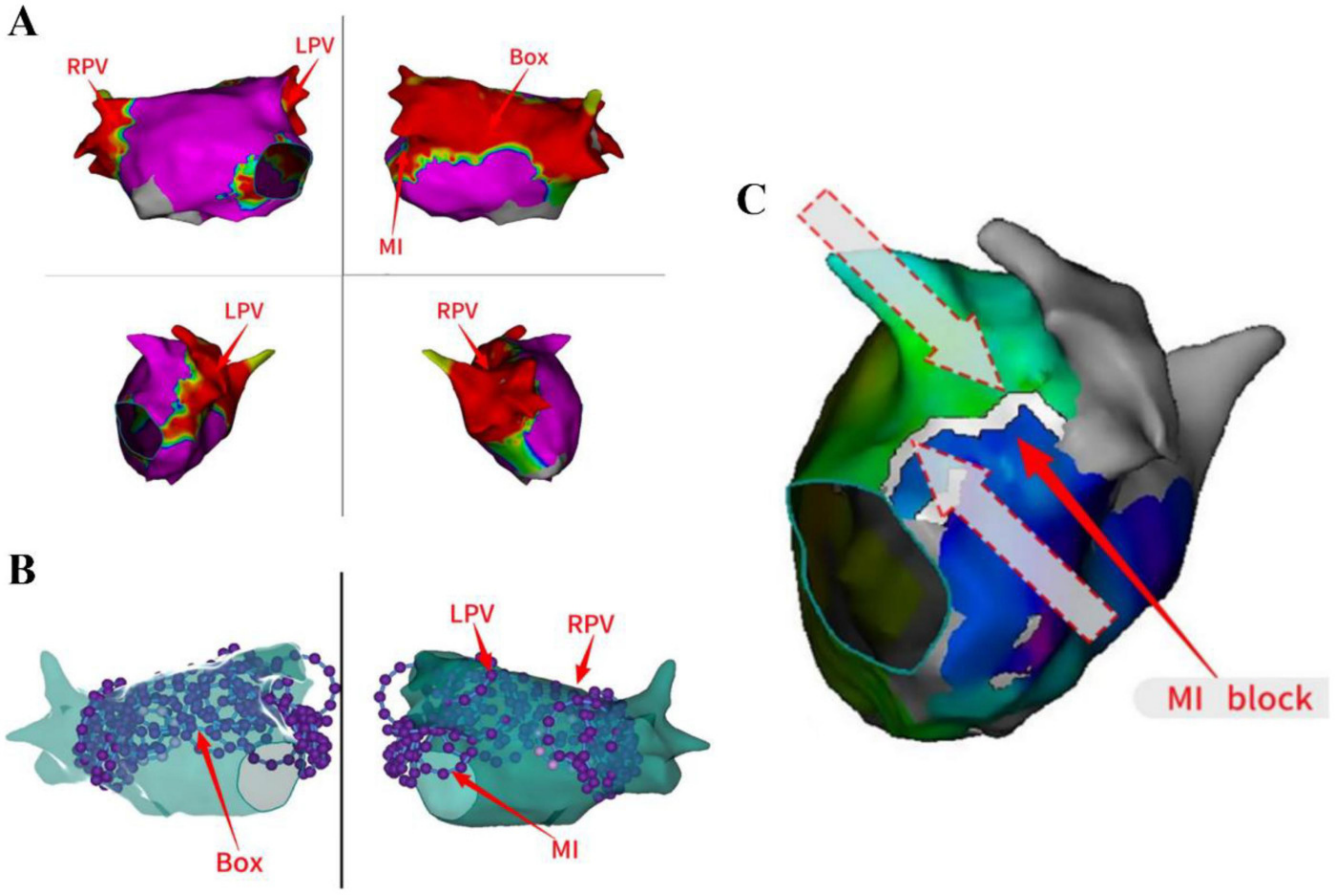
The ablation lines in the PVVs and MI were appeared distinct. The ablation line within the pulmonary vein antrum and mitral isthmus vestibule appeared distinct **(A–C)**. MI, mitral isthmus; LPV, Left pulmonary vein; RPV, right pulmonary vein.

The patient exhibited signs of AF, and our initial strategy involved undertaking left atrial substrate mapping while the AF remained ongoing (Figures 1A,B). We successfully ablated the pulmonary venous vestibule and achieved electrical isolation of the pulmonary vein's potential, each pulmonary vein was isolated with a series of 4 ablation lesions. Then linear ablation was performed from the posterior mitral annulus to the anterior aspect of the left inferior pulmonary vein orifice, utilizing a steerable sheath to ensure stable catheter contact. After completion of pulmonary vein potential isolation and mitral isthmus line ablation, AF was converted into atrial flutter. The atrial flutter exhibited a cycle length of 170 ms and demonstrated eccentric conduction with early CS1-2 polar atrial waves (Figure 1C). Atrial flutter was confirmed to be a mitral isthmus-dependent atrial flutter. Therefore, we continued to strengthen mitral isthmus ablation. After this ablation procedure, the atrial flutter presented a cycle length of 210 ms and exhibited conduction marked by early CS9-10 polar atrial waves (Figures 1D,E). Then we performed BOX ablation on the posterior wall of the left atrial. The entire posterior wall was ablated with a total of 8 overlapping lesions. Stable catheter contact and a “kissing” configuration between poles 1 and 10 were confirmed. The circular ablation pattern ensured contiguous lesion sets with ring overlap, analogous to the interconnected rings of the “Audi” logo (Figure 1F). After the left atrial ablation was completed, the ablation line within the pulmonary vein antrum and mitral isthmus vestibule appeared distinct (Figures 2A–C).

**Figure 3:**
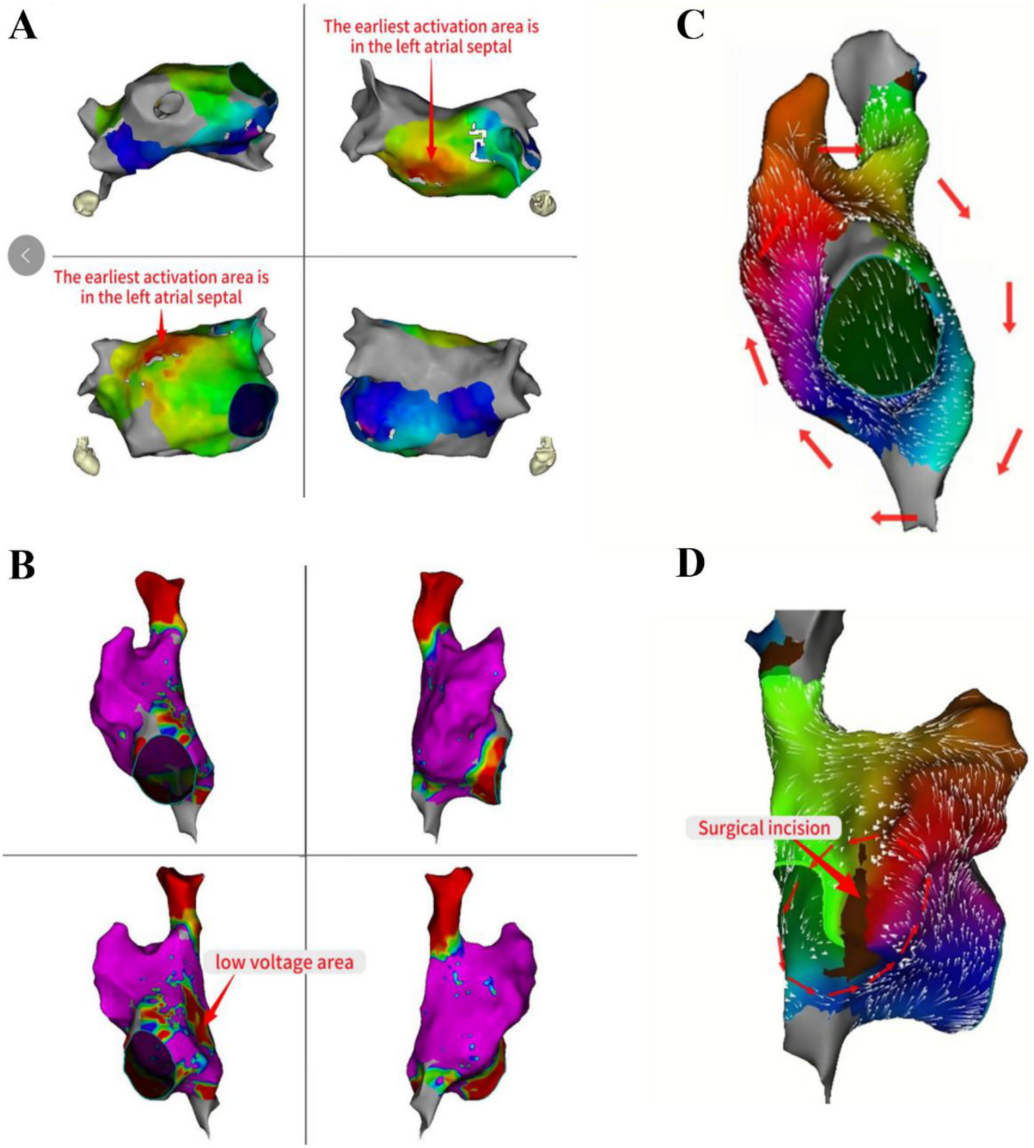
Mapping of left atrial activation patterns and right atrial dual reentrant circuits. The propagation pattern in the left atrial revealed that the atrial septum was excited earlier **(A)**. The right atrial substrate mapping clearly showed a low-voltage area in the right atrial **(B)**. The activation mapping revealed the two reentrant loops in the right atrial, one traveled around the tricuspid annulus, and the other around the surgical incision **(C,D)**.

**Figure 4:**
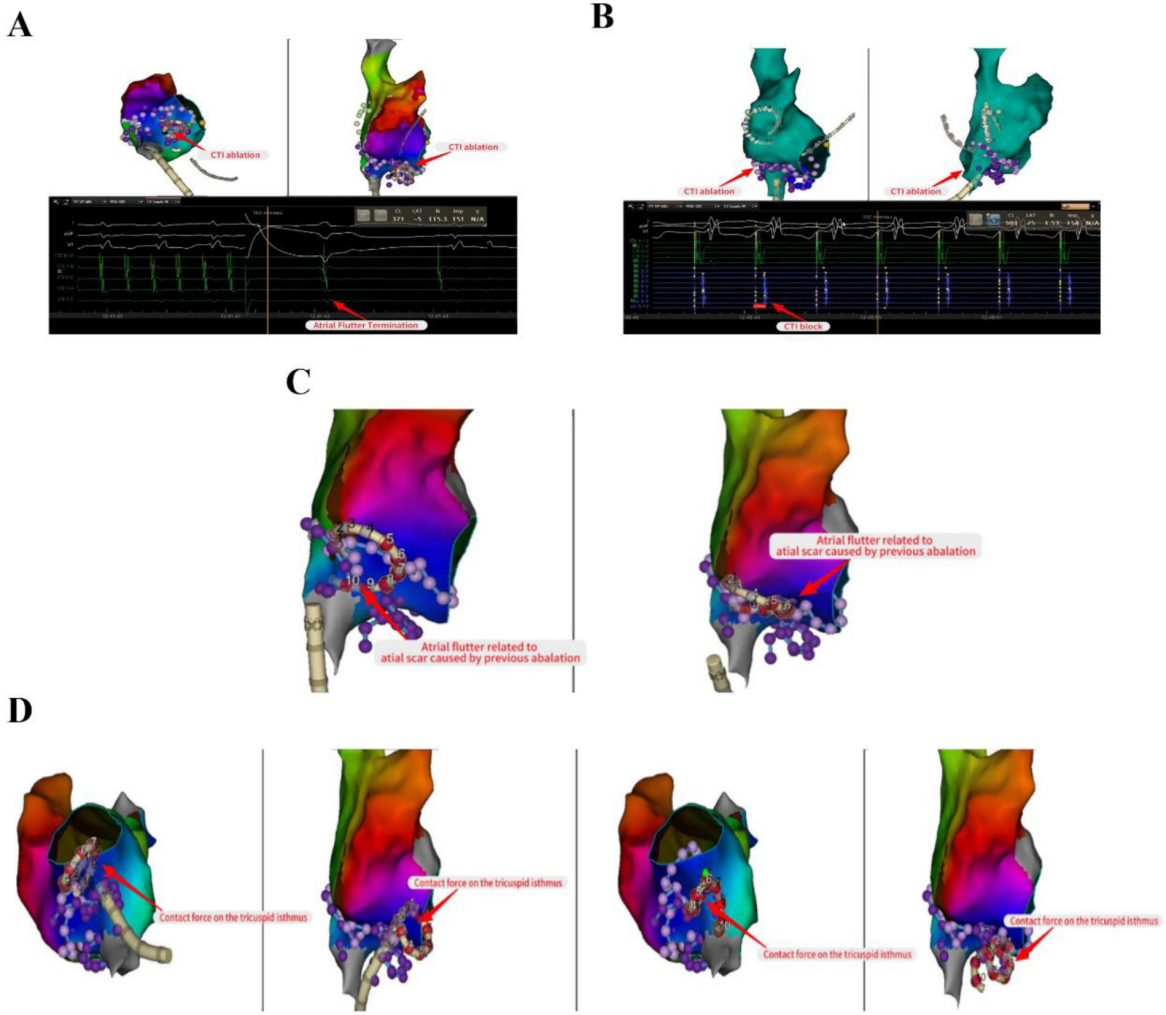
Linear ablation of tricuspid isthmus and surgical incision. Ablated the tricuspid isthmus to halt atrial flutter and validated the tricuspid isthmus line block after the surgical procedure **(A,B)**. Continued linear ablation along the incision towards the inferior vena cava **(C)**. The series of images comprehensively illustrates the technique of the VARIPULSE™ catheter adhesion to the isthmus of the tricuspid valve **(D)**.

The propagation pattern in the left atrial revealed that the atrial septum was excited earlier (Figure 3A). Based on the early onset features of atrial flutter in Cs9-10, we decided to persist with mapping the propagation in the right atrial. The right atrial substrate mapping clearly showed a low-voltage area in the right atrial (Figure 3B). The activation mapping revealed the presence of two reentrant loops in the right atrial, one of which traveled around the tricuspid annulus, and the other around the surgical incision (Figures 3C,D). We ultimately ablated the tricuspid isthmus to halt atrial flutter and validated the tricuspid isthmus line block after the surgical procedure (Figures 4A,B). Complete block was achieved through segment-by-segment ablation using a steerable sheath to ensure electrode contact. Due to the history of atrial septal defect repair, mapping of right atrial excitation revealed atrial flutter excitation around the incision fold, leading to continued linear ablation along the incision towards the inferior vena cava (Figure 4C). Following the completion of ablation, atrial burst pacing and pharmacological provocation with isoproterenol injection were performed, and the patient had no recurrence of arrhythmia. All ablations above were performed with a uniform energy setting of 1800 V. The series of images comprehensively illustrates the technique of the VARIPULSE™ catheter adhesion to the isthmus of the tricuspid valve (Figure 4D).

Postoperatively, the patient persisted in sinus bradycardia, and thus no antiarrhythmic agents were administered. Given the patient's relatively high CHA₂DS₂-VASc score, dabigatran etexilate was prescribed at a dosage of 110 mg twice daily for 3 months.

At the 3-month postoperative follow-up, the patient had no recurrence of arrhythmias such as AF or atrial flutter, and her palpitations were completely relieved.

## Discussion

Linear ablation serves as a pivotal adjunct to catheter ablation for atrial fibrillation, offering substantial therapeutic value in persistent or complex cases (4, 8, 9). While conventional cryoablation and radiofrequency ablation present inherent limitations, PFA effectively addresses the procedural challenges of “technical difficulty and high risk” in anatomically complex regions (3, 6). As an emerging modality utilizing irreversible electroporation to induce myocardial necrosis, PFA demonstrates superior tissue selectivity, enhanced safety profiles, improved procedural efficiency, and broader clinical applicability. Its efficacy in pulmonary vein isolation (PVI) has been extensively validated (10).

The rapid evolution of PFA technology has catalyzed the development of numerous innovative catheters specifically designed for linear ablation. These catheters achieve selective myocardial ablation through precisely controlled high-voltage electrical pulses, showing particular promise in linear ablation pathways for atrial flutter (e.g., cavotricuspid isthmus-dependent flutter) and complex atrial fibrillation substrates (11, 12).

Although conventional perception has favored linear catheters for linear ablation procedures, this case demonstrates that innovative solutions like the Varipulse™ variable-loop catheter represent breakthrough technologies. Featuring an adaptive circular geometry, this dynamic design enables real-time catheter curvature adjustment and achieves comparable effectiveness and safety to linear catheters through segmented contact patterns, while demonstrating superior efficiency in complex scenarios combining PVI with linear lesions (10, 13, 14).

For anatomical variations including the mitral isthmus curvature, cavotricuspid isthmus pathway, and post-ablation scar topography, this technology holds potential for optimizing the entire procedural workflow—encompassing precise positioning, dynamic adaptation, and efficient ablation.

## Conclusions

The Varipulse™ catheter's design enables real-time curvature adjustment to accommodate anatomical variations, thus ensuring favorable reliability and safety in atrial fibrillation linear ablation.

## Data Availability

The original contributions presented in the study are included in the article/Supplementary Material, further inquiries can be directed to the corresponding author/s.
